# An Invasive Plant Promotes Its Arbuscular Mycorrhizal Symbioses and Competitiveness through Its Secondary Metabolites: Indirect Evidence from Activated Carbon

**DOI:** 10.1371/journal.pone.0097163

**Published:** 2014-05-09

**Authors:** Yongge Yuan, Jianjun Tang, Dong Leng, Shuijin Hu, Jean W. H. Yong, Xin Chen

**Affiliations:** 1 College of Life Sciences, Zhejiang University, Hangzhou, China; 2 Department of Plant Pathology, North Carolina State University, Raleigh, North Carolina, United States of America; 3 Singapore University of Technology and Design, Singapore, Singapore; University of Tartu, Estonia

## Abstract

Secondary metabolites released by invasive plants can increase their competitive ability by affecting native plants, herbivores, and pathogens at the invaded land. Whether these secondary metabolites affect the invasive plant itself, directly or indirectly through microorganisms, however, has not been well documented. Here we tested whether activated carbon (AC), a well-known absorbent for secondary metabolites, affect arbuscular mycorrhizal (AM) symbioses and competitive ability in an invasive plant. We conducted three experiments (experiments 1–3) with the invasive forb *Solidago canadensis* and the native *Kummerowia striata.* Experiment 1 determined whether AC altered soil properties, levels of the main secondary metabolites in the soil, plant growth, and AMF communities associated with *S. canadensis* and *K. striata*. Experiment 2 determined whether AC affected colonization of *S. canadensis* by five AMF, which were added to sterilized soil. Experiment 3 determined the competitive ability of *S. canadensis* in the presence and absence of AMF and AC. In experiment 1, AC greatly decreased the concentrations of the main secondary metabolites in soil, and the changes in concentrations were closely related with the changes of AMF in *S. canadensis* roots. In experiment 2, AC inhibited the AMF *Glomus versiforme* and *G. geosporum* but promoted *G. mosseae* and *G. diaphanum* in the soil and also in *S. canadensis* roots. In experiment 3, AC reduced *S. canadensis* competitive ability in the presence but not in the absence of AMF. Our results provided indirect evidence that the secondary metabolites (which can be absorbed by AC) of the invasive plant *S. canadensis* may promote *S. canadensis* competitiveness by enhancing its own AMF symbionts.

## Introduction

Why invasive plants have increased competitive ability at their introduced range is the central question in understanding the mechanism of successful plant invasion [1]–[3]. Published studies have shown that invasive plants enhanced their competitive ability via the production of secondary metabolites [4], [5]. Secondary metabolites can mediate invasive plants’ competition via strong effects on nutrient cycling [6], native plants [7], and soil microbes [8]. These influences could be mediated through directly and indirect ways.

The direct mediation of secondary metabolites from invasive plants on plant-plant competition can be best illustrated by observing native plants inhibiting their neighboring plants [9], [10]. For example, roots exudes phytotoxin (−)-catechin from the invasive *Centaurea maculosa* directly inhibited the growth of native plants [7]. Secondary metabolites released from other invasive plants also directly inhibited seed germination of native plants [9], [10]. The inhibition resulting from such direct effects can obviously increase the competitive ability of the invasive plant [11].

Secondary metabolites released by invasive plants also mediate competition indirectly. For example, secondary metabolites can indirectly promote the growth of invaders via influencing the nutrient cycling, like the pools and fluxes of inorganic and organic soil nutrient [6], or indirectly mediate competition via native soil microorganisms. Plant pathogens and symbionts [12]–[17], and the soil community [18]–[20] can be altered by secondary metabolites in general. Such effects on soil microorganisms may alter the competition between invasive and native plants [21].

Arbuscular mycorrhizal fungi (AMF) are important and ubiquitous soil microorganisms that form symbioses with many plant species in terrestrial ecosystems [22], [23]. Interestingly, AMF also influence invasive plants’ competition [24], [25]. For example, invasive Asian knapweed (*Centaurea maculosa*) in North America is able to tap into and benefit from the mycorrhizal network linking native plant roots [26], [27]. Invasive *Solidago canadensis* in China can alter the AMF composition in the introduced range in ways that promote its competition against native plants [20]. In some cases, the secondary compounds released by invasive plants disrupt the symbioses between the AMF and native plants. For example, secondary chemicals released by the invasive plant *Alliaria petiolata*, which cannot form symbioses with the AMF, disrupted the symbioses between native canopy tree seedlings and AMF [28]. A specific flavonoid fraction released by *A. petiolata* has a far stronger inhibitory effect on AMF in the invaded soil than in its original soils [29]. Additionally, the secondary compounds from *A. petiolata* not only inhibited AMF hyphal growth and spore germination [30] but also altered the AMF community associated with native sugar maple seedlings [31].

Although substantial evidence demonstrates that invasive plants can affect AMF symbioses of local hosts via secondary metabolites [30], [32]–[36], whether those secondary metabolites can affect symbioses between AMF and the invasive plants themselves has not been documented. We address this question by using activated carbon (AC) to absorb secondary metabolites. Generally the effect of AC addition on plant growth and the formation of mutualistic relationships can reflect the putative role of secondary metabolites [37].

We used the perennial herb *Solidago canadensis* L. (goldenrod) as a model plant in our study. *S. canadensis* is native to North America but forms a near monoculture in areas of China where it has invaded [38]. *S. canadensis* has a strong allelopathic effect on native plants and is always associated with AMF, making it suitable for testing our hypothesis [39]. In a previous study, we found that the concentrations of three main secondary metabolites in *S. canadensis* plants were greater in the invaded range than in its native range and that these compounds substantially increased the competitive ability of *S. canadensis* in the invaded range [11]. Experiments have also shown that invasive *S. canadensis* is always associated with AMF and can alter the AMF community in ways that enhance its competitive ability [20], [40]. In the current study, we separated the indirect effects (i.e., those mediated by AMF) and direct effects (i.e., those not mediated by AMF) of secondary metabolites from *S. canadensis* on the competition between *S. canadensis* and a native plant.

## Materials and Methods

### Activated Carbon

Activated Carbon (AC) is often used as an experimental tool to absorb secondary metabolites in soil [41]–[43]. Researchers have noted, however, that even in the absence of secondary metabolites, AC can affect plant growth by altering nutrient availability and other soil properties [44], [45]. In a preliminary experiment, we tested whether AC affected soil properties with and without plants. There were five replicates for each treatment. The experiment lasted for six months. Soil samples were air-dried and sieved carefully, prior to testing. The concentration of soil organic matter (SOM) was measured by the K_2_CrO_4_–H_2_SO_4_ oxidation method of Walkey and Black [46]. Total nitrogen (TN) and total phosphorus (TP) in soil samples (1.0 g per sampling treatment) was determined with a San++ Continuous Flow Analyzer (Skalar, Netherlands). The pH of the soil from each sampling plot was measured using a soil–water slurry (1∶5 soil:water). Soil available phosphorus (AP) was analyzed by the Olsen method [47]. AC at a rate of 20 ml l^−1^ did not affect soil properties (Table 1), and we therefore used AC at 20 ml l^−1^ in this study. The P and N concentration in the AC was 0 and 0.18%, respectively, and the pH was 6.0. The effect of AC on soil properties was also evaluated in experiment 1 of the current study, as described later.

**Table 1:** Effects of AC treatments on soil properties with and without plants.

	Without plants			With plants		
Soil property	No AC	AC	significance	No AC	AC	significance
pH	8.18	8.19	ns	7.96	8.03	ns
TN (g/kg)	0.55	0.56	ns	0.58	0.61	ns
TP (g/kg)	0.62	0.63	ns	0.49	0.50	ns
SOM (g/kg)	12.23	13.25	ns	12.34	11.80	ns
AP (mg/kg)	21.87	21.08	ns	18.06	18.69	ns

TN refers to total nitrogen; TP refers to total phosphorous; SOM refers to soil organic matter; AP refers to available phosphorous. No effect was significant at *P*<0.05. ns, not significant.

### Plant Species

In a previous study with six *S. canadensis* populations (three invasive populations from China and three native populations from America) and one *K. striata* population (from the area invaded by *S. canadensis* in China), we determined that the enhanced competitive ability of *S. canadensis* relative to *K. striata* in China resulted from increased root exudates [11]. Because neither biomass nor height of *S. canadensis* in our previous study was significantly affected by the source of the *S. canadensis* population (either from the original or invaded lands), the current study used one invasive *S. canadensis* population originating from the invaded area in China. One native *K. striata* population from China was also used. Seeds of *S. canadensis* and *K. striata* were collected in the same locations (30°16′N, 120°11′E) in Hangzhou, Zhejiang province (China), where the area was invaded by *S. canadensis*. Seeds of *S. canadensis* and *K. striata* were air-dried and stored at 4°C.

### Experiment 1

A greenhouse microcosm experiment was performed to determine whether AC affects soil properties, the levels of three groups of secondary metabolites (total flavones, total phenolics, and total saponins) in soil, plant growth, and the AMF community associated with invasive *S. canadensis* and native *K. striata.*

The experiment was a two factorial design, with two AC treatments (with and without AC), two host plants (invasive *S. canadensis* and native *K. striata*), and five replicates (five blocks). Each microcosm measured 20×15×20 cm (length×width×height) and contained 6 kg of soil. The soil used in the experiment was collected from Cixi City, Zhejiang Province, China (30°18′N, 121°10′E), where *S. canadensis* had invaded the locality.

To ensure the initial consistency of plants in the experiment, we germinated and pre-cultured the plants in a plastic mesh plate with vermiculite and peat in the greenhouse with natural light and temperature [11]. When seedlings were 4 cm tall, two seedlings were transplanted into each microcosm so that the microcosm contained two *S. canadensis* seedlings or two *K. striata* seedlings. For the AC treatment, finely ground AC was mixed into the soil at 20 ml l^−1^ [11].

Microcosms were randomly arranged in each block in a greenhouse with natural light and with average daily temperatures ranging from 18–30°C during the experiment, which began in April and ended in October. Each microcosm was watered daily to maintain soil moisture at 70–90% of water-holding capacity. No additional nutrients were added during the experiment.

Experiment 1 was terminated 6 months after transplanting, which coincided with floral initiation. Root systems were separated from shoots. Half of each root sample was frozen at −80°C for molecular analysis. The remaining half of each root sample was used for quantification of AMF colonization. AMF colonization of roots was quantified using a microscope (×20 magnification) and utilizing the gridline intersection method [48], and 200 transects were examined per replicate.

Soil samples in each microscope were air-dried and sieved for the test of soil properties. Soil properties were measured with the methods described in the preliminary experiment. Soil organic matter (SOM), total nitrogen (TN), total phosphorus (TP), available phosphorus (AP) and pH were tested. Three main secondary metabolites (total flavones, total phenolics, and total saponins) in the soil were also quantified using the methods described by Zhang et al. [49]. First, the crude soil extracts were obtained by placing the 10-g sample in 100 ml of 70% ethanol and later, filtering the sample through a Whatman no. 44 filter paper. Following which, total flavones in the crude extracts were determined by employing the NaNO_2_–Al(NO_3_)_3_–NaOH colorimetric assay with rutin as the reference substance. Total phenolic acids were measured by the Folin-Ciocalteu assay with gallic acid as the reference substance. The total saponins were detected using vanillin–HClO_4_ as the chromogenic reagent.

To examine the effects of AC on AMF in roots of *S. canadensis* and *K. striata*, we used a nested PCR-denaturing gradient gel electrophoresis (DGGE)-cloning-sequencing method. Each plant species was represented by three replicates. The details of the methods are described in Methods S1. The DGGE banding pattern obtained using this method was analyzed with Quantity-One software (Bio-Rad).

Each DNA sequence obtained from the AMF was subjected to similarity comparison using an online program (BLAST, http://www.ncbi.nlm.gov/BLAST). Sequences of possible chimeric origin were detected using the online CHIMERA DETECTION program (http://rdp8.cme.msu.edu/html/analyses.html). Sequences were subsequently registered in the GenBank database under accession numbers KC507871–KC507891. For the phylogenetic analysis, sequences with gaps were treated as missing data, and then a neighbor-joining tree (1,000 replicates) including the obtained sequences and their closest relatives from GenBank was constructed using MEGA version 4.0 with the Kimura 2-Parameter model. Sequences that were 2% different from each other were subsequently assigned to separate phylotypes. It is noteworthy that the tree topology and bootstrap value were always included in the analysis.

To analyze the potential influence of secondary metabolites and soil properties on the AMF community composition, we performed a canonical correspondence analysis (CCA) with Monte-Carlo permutation tests (n = 499) using the Canoco for Windows version 4.52. In the CCA plot, the length of the arrows illustrated the relative importance of factors affecting community structure, while the angle between the arrows indicated the degree to which factors are correlated.

The presence (or absence) and intensity of the bands in a DGGE profile were used to construct a two-dimensional matrix. Two-way analysis of similarity (ANOSIM) was performed using the Past, version 1.91 program [50] to analyze AMF communities differences subjected to different AC treatments.

After the data for soil properties, shoot biomass, and AMF colonization were tested for normality and homogeneity of variances, we used a one-way ANOVA in the general linear model to determine the effects of AC on soil properties, shoot biomass, and AMF colonization of *S. canadensis* and *K. striata*.

### Experiment 2

This experiment was conducted to study the effects of AC on the symbioses between *S. canadensis* and five AMF species respectively. In contrast to experiment 1, experiment 2 used sterilized soil that was subsequently inoculated with selected AMF. Based on the results from experiment 1 and the experiments of Zhang et al. [20], which indicated that *Glomus* was the dominant AMF genus in *S. canadensis* roots, we used the following five *Glomus* species in experiment 2: *G. mosseae* (BGC501, XJ-01), *G. versiforme* (BGC504, BJ08), *G. diaphanum* (BGC506, SC05), *G. geosporum* (BGC507, GZ01), and *G. etunicatum* (BGC505, TW01). The AMF species were provided by the Glomales Germplasm Bank in China. These five *Glomus* species existed naturally in abandoned fields where the seeds of *S. canadensis* and *K. striata* were collected [20], [51]. Soil with spores was used as the inoculum for the experiment.

Experiment 2 employed the following experimental design: two factors (AC and AMF) with five replicates (five blocks). The soil was the same as in experiment 1. AC was added to half of the microcosms at 20 ml l^−1^ to absorb secondary compounds. After each microcosm was filled with 3 kg soil that had been sterilized by γ-radiation, an equal number of spores of each AMF species was mixed into the soil (one species per microcosm).

Seeds of *S. canadensis* were germinated and pre-cultured as described in experiment 1. Two seedlings were transplanted into each microcosm. The microcosms were randomly arranged in each block in the greenhouse. All growth conditions were the same as in experiment 1. Six months after transplanting, the plants were harvested, and roots were separated from shoots. Shoot and root samples were oven-dried (65°C for 48 h) for the measurement of dry total biomass. AMF colonization of roots was quantified as described for experiment 1. Spores were separated from the soil by the wet-sieving method and counted using a microscope [52].

The effects of AC on total biomass of *S. canadensis*, AMF colonization and spore numbers in soil were determined with a two-way ANOVA in the general linear model after the data had been checked for normality and homogeneity of variances. When ANOVAs were significant, mean values were compared by least significant difference (LSD) at the 5% significance level.

### Experiment 3

Experiment 3 examined how AC altered the competitive ability of *S. canadensis* in the presence and absence of AMF. The AMF inoculum was a mixture of three *Glomus* species: *G. intraradices*, *G. mosseae*, and *G. geosporum*. The three AMF species were also provided by the Glomales Germplasm Bank in China.

The experiment was a three factorial design, with two AC treatments (No AC and AC), two AMF treatments (AMF and No AMF), and three plants culture types (monoculture of invasive, monoculture of native, and a mixture of invasive and native), and five replicates (five blocks). A total of 2×2×3×5 = 60 microcosms were produced. The microcosms and soil were the same as in experiment 1. Each microcosm was filled with 6 kg of soil that had been sterilized by γ-radiation and subsequently inoculated or not inoculated with AMF. Control microcosms without AMF received equal amounts of AMF inoculum sterilized by γ-radiation plus filtrate from the AMF inoculum that contained no AMF propagules; this was done to provide experimental control to account for potential mineral and non-mycorrhizal microbial components in the inoculum. AC was added to half the microcosms as indicated in experiment 1.

Two *S. canadensis* seedlings, two *K. striata* seedlings, or one *S. canadensis* seedling and one *K. striata* seedling were transplanted into each microcosm. The microcosms were randomly placed in each block in the greenhouse. All growth conditions were the same as in experiment 1.

The plants were harvested after 6 months, just before they began to flower. Roots were separated from shoots. Shoot samples were oven-dried (65°C for 48 h) for measurement of dry shoot biomass. AMF colonization of roots was quantified as described earlier for experiment 1. The competitive ability of *S. canadensis* was determined using an aggressivity index (AI), which was based on shoot biomass data. The AI was calculated as described previously [11], [53]. AI = (Y_ij_/Y_ii_)−(Y_ji_/Y_jj_), where Y_ij_ and Y_ii_ are the shoot biomasses of *K. striata* growing in mixture and monoculture, and Y_ji_ and Y_jj_ are the shoot biomasses of *S. canadensis* growing in mixture and monoculture. The competitive ability of *S. canadensis* relative to *K. striata* is inversely related to the AI value.

After the data for shoot biomass and AMF colonization were checked for normality and homogeneity of variances, we used a two-way ANOVA in the general linear model to test the effect of AC and AMF on shoot biomass and AMF colonization of *S. canadensis* and *K. striata* in monoculture or in mixture.

After checking the AI data for homogeneity, we used a two-way ANOVA in the general linear model to examine the effects of AC and AMF on AI. Mean values were compared by least significant difference (LSD) at the 5% significance level.

## Results

### Effects of AC on Soil Properties, Plant Growth, and AM Symbiosis (Experiment 1)

AC did not affect the key attributes of soil properties (soil organic matter, total nitrogen, total phosphorus, available phosphorus and pH) (*P*>0.05) but significantly decreased the levels of the main groups of secondary compounds (total flavones, total phenolics, and total saponins) (*P*<0.01) in soil planted with *S. canadensis* or *K. striata* (Table 2).

**Table 2:** Effects of AC treatments and plants (invasive *S. canadensis* or native *K. striata*) on soil properties in experiment 1.

	*S. canadensis*			*K. striata*		
Soil property	No AC	AC	significance	No AC	AC	significance
pH	7.77	7.78	ns	7.77	7.77	ns
TN (g/kg)	1.03	1.01	ns	1.02	1.05	ns
TP (g/kg)	0.50	0.48	ns	0.49	0.50	ns
SOM (g/kg)	20.54	23.04	*	20.34	21.20	ns
AP (mg/kg)	49.02	48.57	ns	48.06	48.69	ns
Tph (ppm)	204.40	5.61	**	107.81	2.24	**
Tfl (ppm)	47.67	0.48	**	23.50	0.17	**
Tsa (ppm)	467.67	23.80	**	231.00	19.9	**

TN refers to total nitrogen; TP refers to total phosphorous; SOM refers to soil organic matter; AP refers to available phosphorous; Tph refers to total phenolics; Tfl refers to total flavones; Tsa refers to total saponins. For each plant species, a significant difference between No AC and AC is indicated as follows:

*P<0.05;

**P<0.01;

ns *P*>0.05 (not significant).

**Figure 1:**
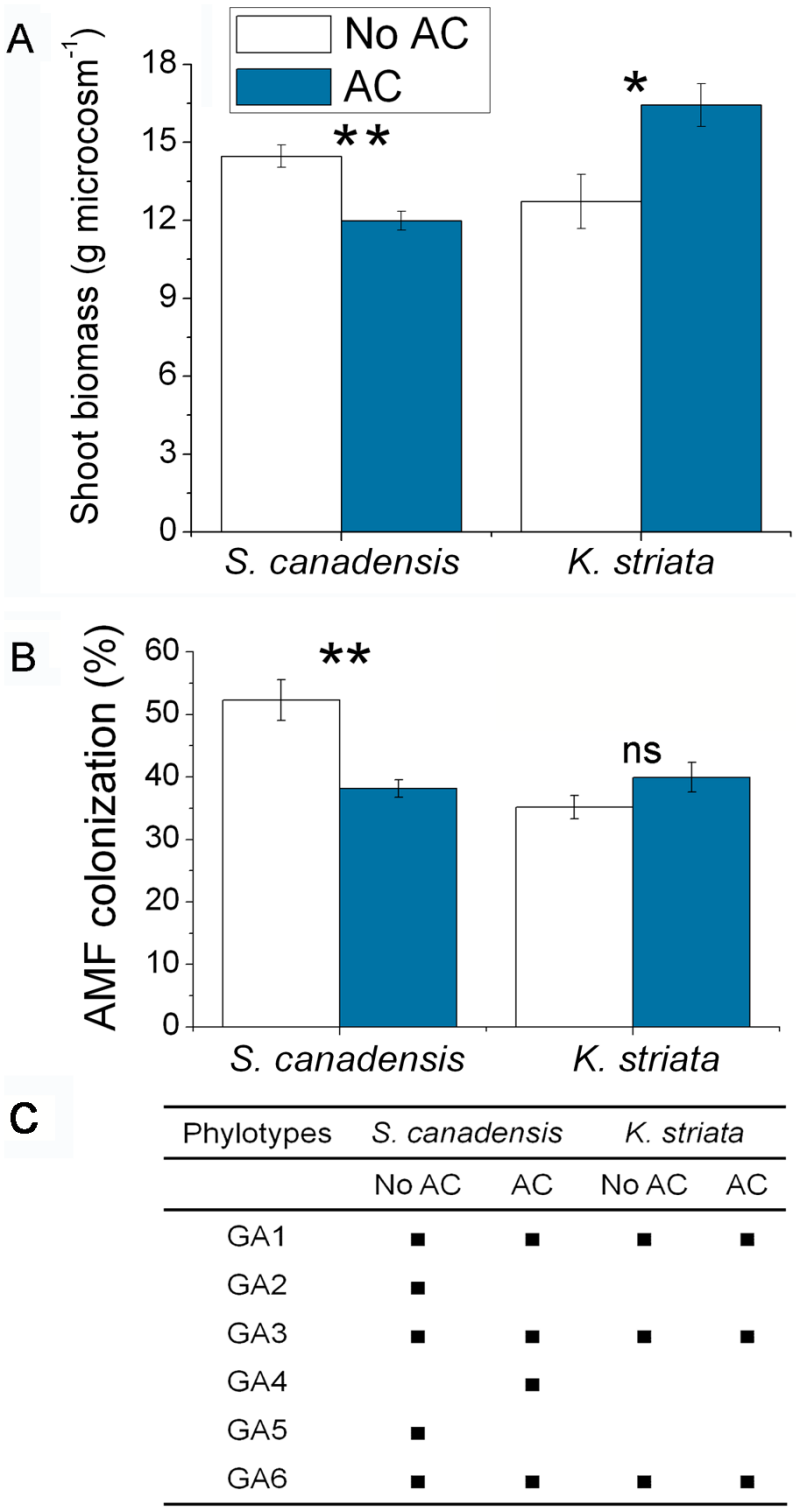
Effects of activated carbon (AC) on shoot biomass and AMF community of plants (experiment 1). Effects of AC on shoot biomass of *S. canadensis* and *K. striata* (A), AMF colonization rate (B) and on the phylotypes of AMF communities in the roots of *S. canadensis* and *K. striata* (C) growing in field soil in experiment 1. GA1–GA6 are phylotypes (Fig. S2). Values are means ± SE. *P*-value: *<0.05; **<0.01; ns, not significant. Means with different letters are significantly different.

The addition of AC to soil significantly reduced the shoot biomass of *S. canadensis* (*F*_1,8_ = 58.169, *P* = 0.007) but increased the shoot biomass of *K. striata* (*F*_1,7_ = 9.561, *P* = 0.018) (Fig. 1A). AC reduced AMF colonization of *S. canadensis* (*F*_1,8_ = 47.287, *P*<0.001) but did not affect AMF colonization of *K. striata* (*F*_1,7_ = 2.523, *P* = 0.131) (Fig. 1B).

DGGE profiles showed that the AMF composition differed in invasive *S. canadensis* versus native *K. striata*. AC affected the pattern and signal intensity of bands of the 18S rDNA fragments of AMF in roots of *S. canadensis* and *K. striata* (Fig. S1). The phylogenetic analysis indicated that sequences retrieved from all root samples could be separated into six AMF groups (GA1–GA6) (Fig. S2). AC treatments affected AMF phylotypes in *S. canadensis* roots but not in *K. striata* roots (Fig. 1C). Roots of *S. canadensis* were colonized by five AMF phylotypes when AC was not added (group GA4 was missing) and by four phylotypes when AC was added (GA2 and GA5 were missing) (Fig. 1C). AMF phylotypes GA1, GA3, and GA6 in roots of *S. canadensis* or *K. striata* were not affected by the addition of AC (Fig. 1C).

The ANOSIM output showed that the AMF communities was significantly different with different AC treatments (*P* = 0.011) and host plants (*P* = 0.010).

**Figure 2:**
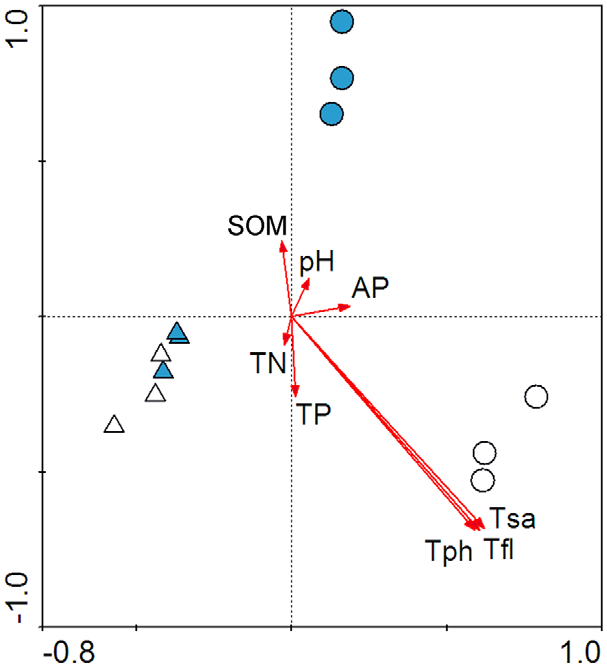
Factors affecting the changes of AMF communities of host plants (experiment 1). CCA-biplot depicting the relationship between AMF communities and soil properties. Circles represent *S. canadensis*, and triangles represent *K. striata*; open symbols indicate No AC treatment while filled symbols indicate AC treatment. TN refers to total nitrogen; TP refers to total phosphorous; SOM refers to soil organic matter; AP refers to available P; Tph refers to total phenolics; Tfl refers to total flavones; Tsa refers to total saponins. The length of the arrows indicates the relative influence of the factor on community structure, while the angle between the arrows indicates the degree to which factors are correlated.

The CCA-biplot showed that AC treatment significantly altered the AMF community structure of *S. canadensis* but not of *K. striata* (Fig. 2). The CCA-biplot also demonstrated that the differences in AMF community structures of *S. canadensis* between the No AC and AC treatments were more closely related to the three groups of secondary compounds (total flavones, total phenolics, and total saponins) than to soil properties.

### Effects of AC on Growth of *S. canadensis* and AMF, when Five AMF Species were Added to Sterilized Soil (Experiment 2)

**Figure 3:**
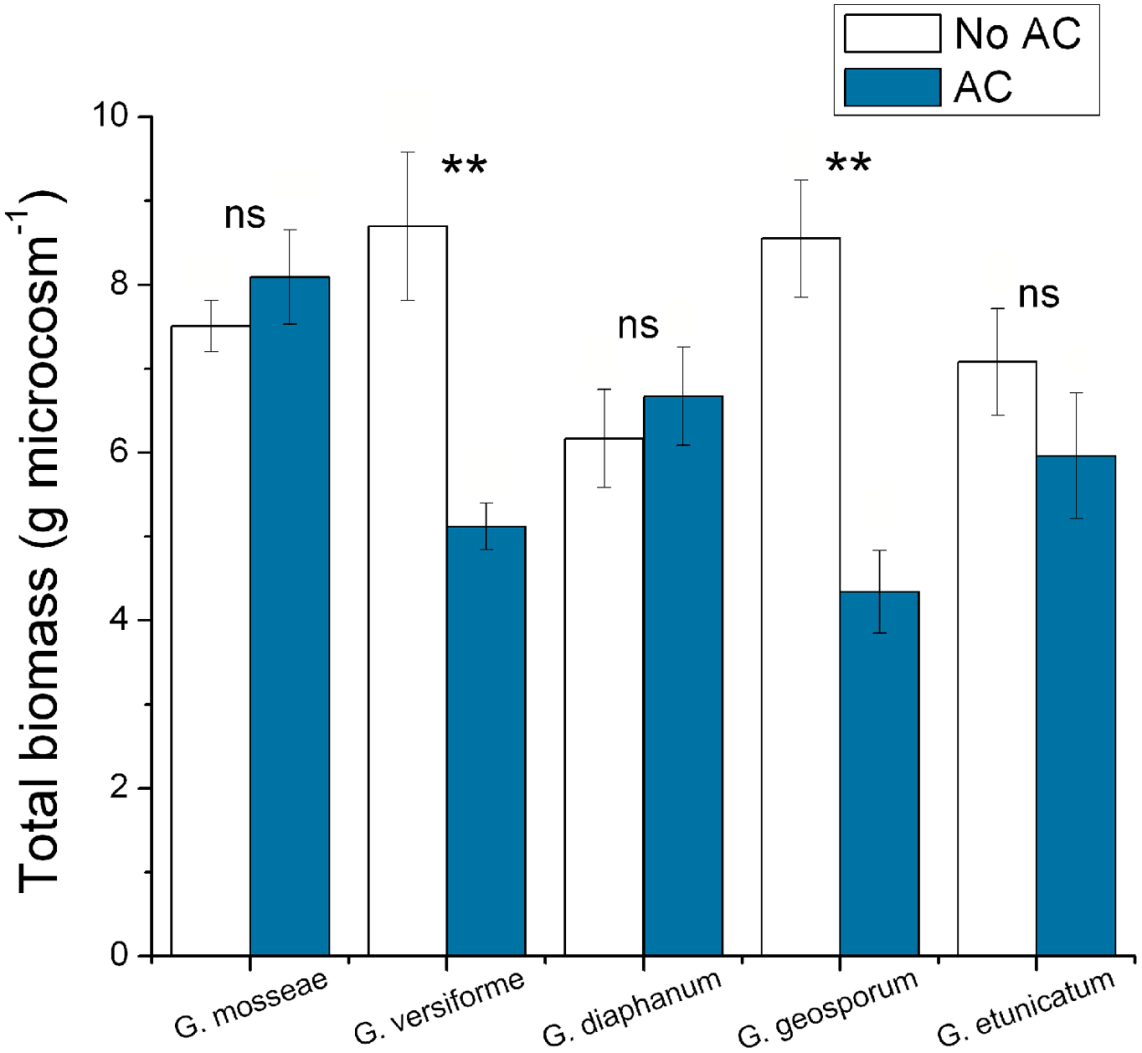
Effects of AC on total biomass of *S. canadensis* (experiment 2). Total biomass of *S. canadensis* inoculated with five AMF species under different activated carbon (AC) treatments in experiment 2. Values are means ± SE. *P*-value: **<0.01; ns, not significant.

AC treatment reduced the total biomass of *S. canadensis* when inoculated with *G. geosporum* and *G. versiforme* (*P*<0.05), but did not affect the total biomass of *S. canadensis* when inoculated with *G. mosseae*, *G. diaphanum* and *G. etunicatum* (*P*>0.05). In the absence of AC, *S. canadensis* attained higher total biomass with *G. geosporum* and *G. versiforme* than with other three AMF species (*G. mosseae*, *G. diaphanum* and *G. etunicatum*) (Fig. 3).

**Figure 4:**
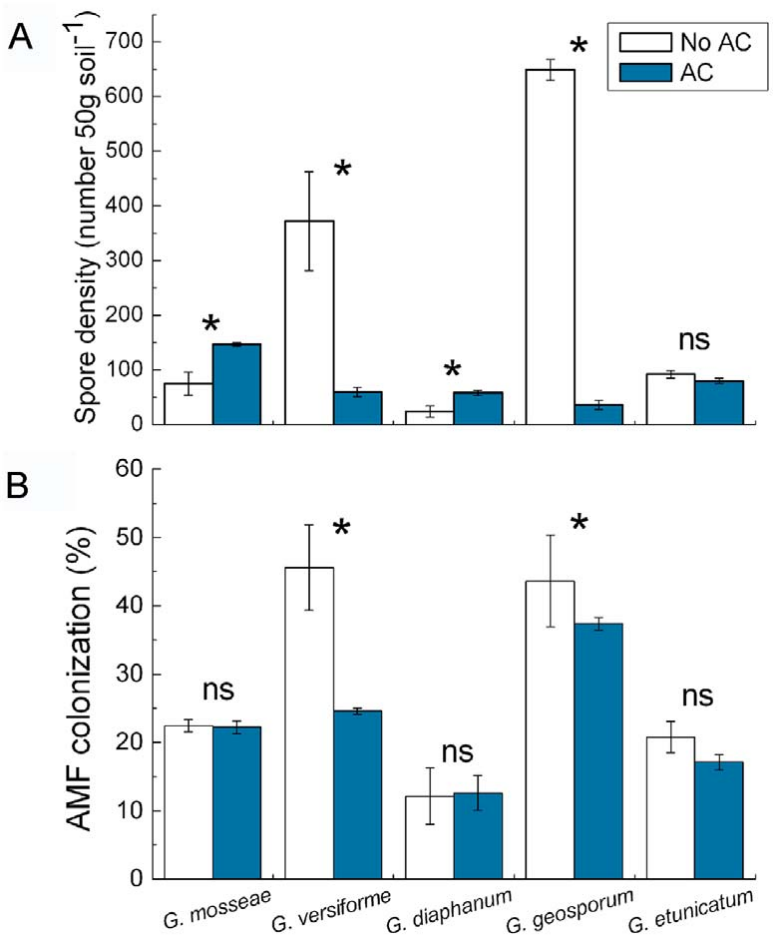
Effects of AC on AMF spore density and colonization of *S. canadensis* (experiment 2). Spore density in soil (A) and colonization of *S. canadensis* roots (B) for five AMF species as affected by addition of activated carbon (AC) in experiment 2. Values are means ± SE. *P*-value: *<0.05; ns, not significant.

Spore density in soil and colonization of *S. canadensis* roots were high for *G. geosporum* and *G. versiforme* in the absence of AC but were reduced (*P*<0.05) when AC was added (Fig. 4). Addition of AC increased (*P*<0.05) the spore density for *G. mosseae* and *G. diaphanum* (Fig. 4A). By contrast, the addition of AC did not significantly affect (*P*>0.05) spore density or colonization for *G. etunicatum* (Fig. 4).

### Effects of AC on the Competition between *S. canadensis* and *K. striata* in the Presence and Absence of AMF in Soil that had been Previously Sterilized (Experiment 3)

**Figure 5:**
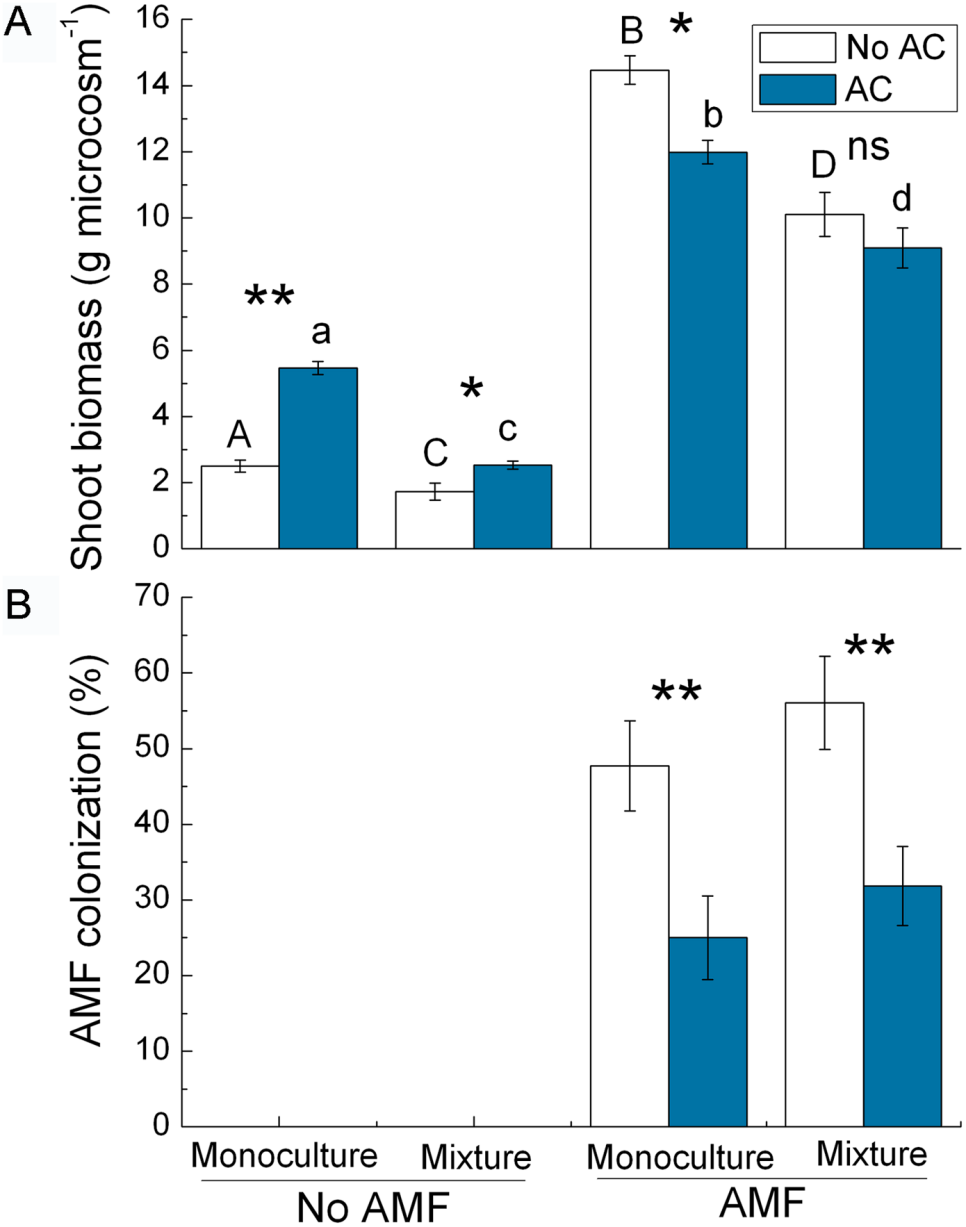
Effects of AMF and AC on growth and AMF colonization of *S. canadensis* (experiment 3). Shoot biomass (A) and AMF colonization (B) of *S. canadensis* under monoculture or mixture (with *K. striata*) as affected by AMF treatments (AMF or No AMF) and activated carbon treatments (AC or No AC) in experiment 3. Values are means ± SE. Means for No AC with different uppercase letters and means for AC with different lowercase letters are significantly different at *P*<0.05. For comparison of paired bars (No AC vs. AC), asterisks indicate significant differences (*<0.05; **<0.01), and ns indicates not significant.

In the absence of AMF, the addition of AC increased *S. canadensis* shoot biomass in monoculture (*F*_1,8_ = 39.526, *P*<0.001) (Fig. 5A) and in mixture (*F*_1,8_ = 8.914, *P* = 0.013) (Fig. 5A). In the presence of AMF, addition of AC decreased *S. canadensis* shoot biomass in monoculture (*F*_1,8_ = 9.429, *P* = 0.017) (Fig. 5A) but did not cause a reduction in biomass when planted in a mixture (*F*_1,8_ = 0.022, *P* = 0.883) (Fig. 5A).

**Figure 6:**
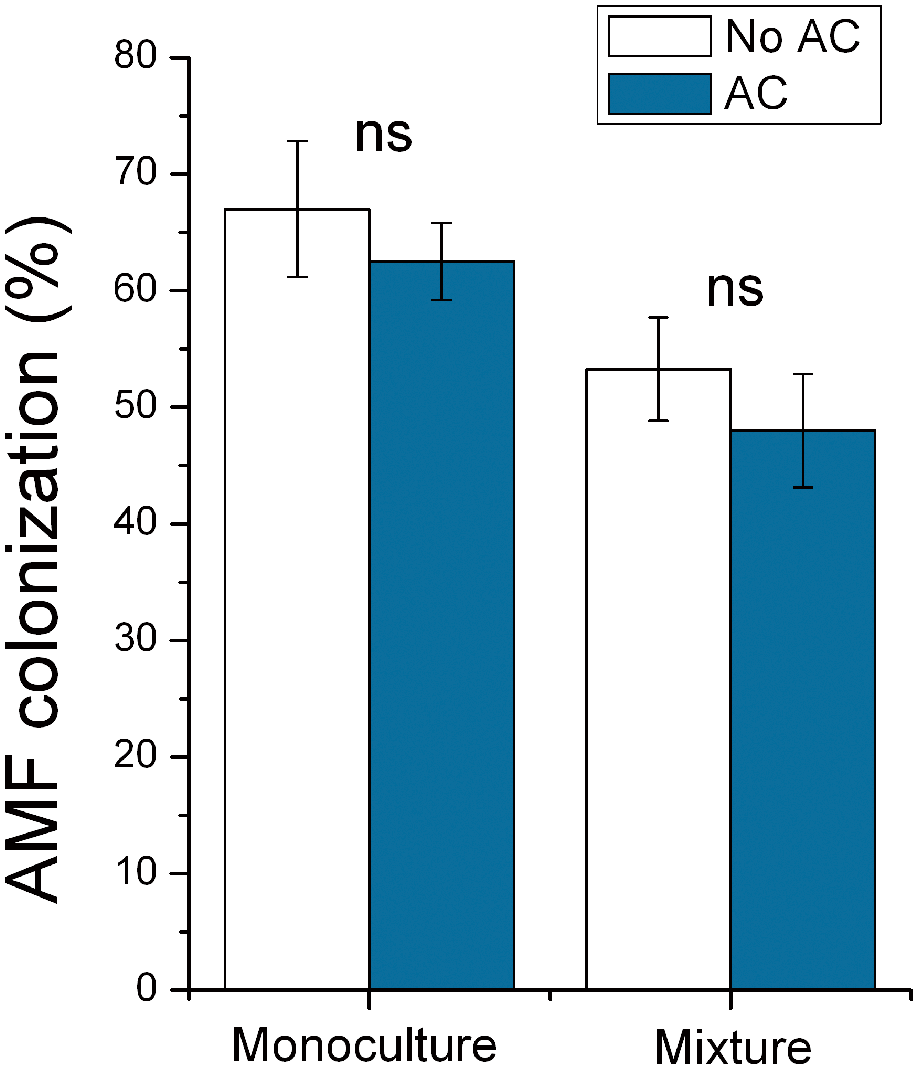
Effects of AC treatments on AMF colonization of *K. striata* (experiment 3). AMF colonization of *K. striata* under monoculture or mixture as affected by AC treatments in experiment 3. Values are means ± SE. For comparison of paired bars (No AC vs. AC), ns indicates not significant.

No AMF spores or any other evidence of AMF colonization were detected in microcosms that were not inoculated with AMF. In soil inoculated with AMF, adding AC reduced AMF colonization of *S. canadensis* in monoculture (*F*_1,8_ = 19.253, *P*<0.001) (Fig. 5B) and in mixture (*F*_1,8_ = 21.452, *P*<0.001) (Fig. 5B) but did not affect AMF colonization of *K. striata* in monoculture (*F*_1,8_ = 0.088, *P* = 0.775) (Fig. 6) and in mixture (*F*_1,8_ = 0.782, *P* = 0.080) (Fig. 6).

**Figure 7:**
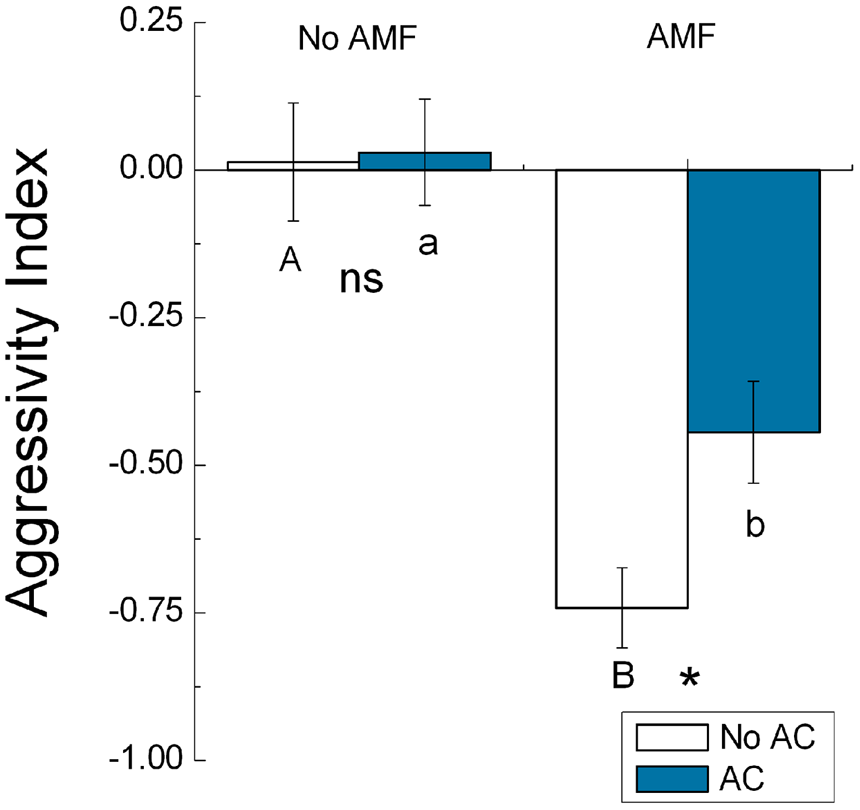
Effects of AMF and AC treatments on the competition of *S. canadensis* (experiment 3). Competitive ability (as indicated by the aggressivity index) of *S. canadensis* as affected by AMF (AMF or No AMF) and activated carbon (AC or No AC) (experiment 3). A lower value for the index indicates greater competitive ability. Values are means ± SE. Means for No AC with different uppercase letters and means for AC with different lowercase letters are significantly different at *P*<0.05. For comparison of paired bars (No AC vs. AC), asterisks indicate significant differences (*<0.05), and ns indicates not significant.

When AMF were not added, AC did not affect the competitive ability (as indicated by AI values) of *S. canadensis* (*F*_1,8_ = 0.023, *P* = 0.745), i.e., the AI values were near zero with or without AC (Fig. 7). When AMF were added, *S. canadensis* AI values were lower (indicating higher competitive ability) without AC than with AC (*F*_1,8_ = 13.776, *P* = 0.012) (Fig. 7). Regardless of AC addition, AI values were much lower with AMF than without AMF (without AC, *F*_1,8_ = 17.851, *P*<0.001; with AC, *F*_1,8_ = 10.415, *P*<0.001) (Fig. 7).

## Discussion

Because activated carbon (AC) absorbs secondary metabolites in soil, it is often used to test the effects of secondary metabolites *in situ* [3], [41]–[43]. AC, however, may also affect nutrient availability [54] and soil physio-chemical properties [45], [55], [56]. Thus, when AC is used in research concerning plant secondary compounds, its putative effects should be checked carefully. In our experiment 1, addition of AC did not change the general soil properties (soil organic matter, total nitrogen, total phosphorus, available phosphorus and pH) but greatly reduced the concentrations of total flavones, phenolics, and saponins. CCA also indicated that variation in the AMF community in *S. canadensis* roots was more closely related to these secondary metabolites than to other soil properties and that these secondary metabolites enhanced the colonization of *S. canadensis* roots by certain AMF species (experiment 2). We therefore deduced that the effects of AC in our study resulted predominantly from the absorption of secondary metabolites by AC.

Our community similarity analysis demonstrated that through the absorption of secondary metabolites, AC affected the AMF community in the roots of *S. canadensis*. Although it was documented that secondary metabolites from invasive plants can affect AM symbiosis of neighboring plants [28], [30], in our study, AC effects suggest that secondary metabolites from an invasive plant may promote its own AM symbiosis and thereby enhancing its own growth.

It remains unclear how plant secondary metabolites affect the process of AMF symbiosis [29], [30], [32], [33]. One plausible avenue could be that some compounds (e.g., flavones, sesquiterpenes, and strigolactones) could act as signals that induce AMF spore germination [57], hyphal branching [32], and/or AM symbiosis formation [36]. Flavones, phenolics, and saponins are commonly produced by many plants [58]–[60]. These three types of compounds had been reported to accumulate in the soil where *S. canadensis* had invaded [49]. Phenolics have been shown to reduce AMF colonization [35], while flavones like chrysin and luteolin can promote AM symbiosis [36]. In this study, AC greatly reduced the levels of three types of secondary metabolites, indicating that the effects of AC on AMF symbiosis in *S. canadensis* may be due to the decrease in the concentrations of secondary metabolites.

Interestingly, not all AMF species were affected in the same way by the secondary metabolites from *S. canadensis*. In experiment 2, for example, without the addition of AC, *S. canadensis* secondary compounds greatly enhanced the growth (as indicated by spore density and root colonization) of *G. geosporum* and *G. versiforme* but did not affect the growth of *G. etunicatum*. The DGGE data and the phylogenetic tree also indicated some AMF phylotypes were present in *S. canadensis* roots only when secondary metabolites had been absorbed by AC. These differential effects on AMF growth confirm that different AMF species can respond differently to the same chemicals. For example, spore germination of *Glomus intraradices* and *G. claroideum* can respond differently to strigolactones [57]. These results suggested that the development of symbioses between specific AMF and *S. canadensis* was mediated by *S. canadensis* secondary metabolites; this selective effect resulted in changes in the AMF community in the roots and also in the soil. In experiment 2, the beneficial AMF species *G. geosporum* and *G. versiforme* were able to promote greater growth of *S. canadensis* than the AMF species that were considered to be less beneficial (Fig. 3). These observations implied that *S. canadensis*’ secondary metabolites (which can be absorbed by AC in the soil) may specifically favor certain compatible AMF partners.

Results from experiment 3 showed that the competitive ability of invasive *S. canadensis* was influenced by both AC and AMF, i.e., release of chemicals by *S. canadensis* enhanced AMF colonization of *S. canadensis* roots. When AMF were absent, secondary metabolites did not increase the competitive ability of *S. canadensis* and autotoxicity might have been generated instead (Fig. 5A). As a consequence, *S. canadensis* grew better with AC treatment than without AC treatment in the absence of AMF. Autotoxicity is also exhibited by other exotic plants, including *Centaurea maculosa* [61], and this phenomenon is a way to reduce intraspecific competition. When AMF inoculum was added to the soil, however, the autotoxic effect was not evident (Fig. 5A), and secondary metabolites enhanced the competitive ability of *S. canadensis* (Fig. 7). These results suggested that AMF can offset the negative effects of secondary metabolites on *S. canadensis*. In other words, secondary metabolites indirectly promoted the competitive ability of *S. canadensis* via AMF. Given that secondary metabolites affected the degree of AMF colonization and the composition of the AMF community in the roots of the invasive *S. canadensis* but had little effect on AMF in the roots of the native *K. striata* in experiments 1 and 3, we concluded that the enhanced competitive ability of *S. canadensis* might have been the consequence of its own secondary metabolites affecting its own AM symbioses.

Secondary metabolites released by an invasive plant may enhance invasiveness by both direct and indirect effects. The direct effect involves the inhibition of native competitors through secondary metabolites without mediation by microorganisms. The indirect effect involves microorganisms that inhibit the native competitors or enhancing the invader [21], [62]. Although both field and common garden experiments have demonstrated that plant secondary metabolites can promote an invader’s competitive ability, past research was unable to separate the direct effects from indirect, microbially mediated effects of invasive non-indigenous plants [14]. It follows that the reported impact of secondary metabolites on competition may in many or all cases have resulted from combined direct and indirect effects. In this study, we separated the direct and indirect effects of secondary metabolites on plant-plant competition using the intrinsic properties of activated carbon. Although our study was performed in microcosms and not in the field, it provided authentic evidence that plant secondary metabolites are able to enhance the competitive ability of an invader by increasing AMF colonization of the invader itself.

In conclusion, the effects of secondary metabolites produced by invasive plants on local plants have been well documented [29], but few studies had focused on how secondary chemicals released by an invasive plant affect that invasive plant itself. Our study demonstrated an interesting phenomenon of “self-promotion” by a plant invader resulting from the positive effects of its own secondary metabolites on the AMF that colonize its roots. The results presented here further expanded our understanding of the interesting role of secondary metabolites during plant invasion.

## Supporting Information

Figure S1 **Effect of AC on DGGE pattern of AMF communities in experiment 1.** DGGE pattern of 18S rDNA fragments of AMF in roots of *S. canadensis* and *K. striata* as affected by addition of activated carbon (AC and No AC) in experiment 1.
(TIF)

Figure S2 **Phylogenetic tree of AMF communities in experiment 1.** Neighbor-joining phylogenetic tree based on partial SSU rRNA gene sequences of all identified AMF and referenced sequences in the root samples. Numbers above branches indicate bootstrap values from 1,000 replicates. The internal identification number represents sequences retrieved from specific DGGE profile bands. A total of 21 partial SSU rRNA sequences were thus obtained in the present study. They are shown in bold and labeled with the GeneBank database accession numbers (i.e., KC507871–KC507891). Sequence groups (GA1, GA2, etc.) identify distinct clusters of sequences with similarity ≥99%.
(TIF)

Methods S1 **Methods used for DNA isolation, PCR amplification, DGGE, cloning, and sequencing analysis of AMF.**
(DOC)

## References

[1] Blossey B, Nötzold R (1995) Evolution of increased competitive ability in invasive nonindigenous plants: a hypothese. Journal of Ecology 83: 887–889.

[2] Siemann E, Rogers WE (2001) Genetic differences in growth of an invasive tree species. Ecology Letters 4: 514–518.

[3] Siemann E, Rogers WE (2003) Increased competitive ability of an invasive tree limited by an invasive beetle. Ecological Applications 13: 1503–1507.

[4] Yang Q, Ye W, Liao F, Yin X (2005) Effects of allelochemicals on seed germination. Chinese Journal of Ecology 24: 1459–1465.

[5] Alford ÉR, Perry LG, Qin B, Vivanco JM, Paschke MW (2007) A putative allelopathic agent of Russian knapweed occurs in invaded soils. Soil Biology and Biochemistry 39: 1812–1815.

[6] Hättenschwiler S, Vitousek PM (2000) The role of polyphenols in terrestrial ecosystem nutrient cycling. Trends in Ecology and Evolution 15: 238–243. 10802549 10.1016/s0169-5347(00)01861-9

[7] Thorpe AS, Thelen GC, Diaconu A, Callaway RM (2009) Root exudate is allelopathic in invaded community but not in native community: field evidence for the novel weapons hypothesis. Journal of Ecology 97: 641–645.

[8] Yu X (2005) A new mechanism of invader success: Exotic plant inhibits natural vegetation restoration by changing soil microbe com-munity. Chinese Science Bulletin 50: 1105.

[9] Ens EJ, Bremner JB, French K, Korth J (2008) Identification of volatile compounds released by roots of an invasive plant, bitou bush (*Chrysanthemoides monilifera* spp. *rotundata*), and their inhibition of native seedling growth. Biological Invasions 11: 275–287.

[10] Cipollini D, Stevenson R, Enright S, Eyles A, Bonello P (2008) Phenolic metabolites in leaves of the invasive shrub, *Lonicera maackii*, and their potential phytotoxic and anti-herbivore effects. Journal of Chemical Ecology 34: 144–152. 18213496 10.1007/s10886-008-9426-2

[11] Yuan Y, Wang B, Zhang S, Tang J, Tu C, et al. (2012) Enhanced allelopathy and competitive ability of invasive plant *Solidago canadensis* in its introduced range. Journal of Plant Ecology. DOI:10.1093/jpe/rts033.

[12] Liu SQ, Wu FZ, Wen XY (2013) Allelopathic effects of root exudates of Chinese onion on tomato growth and the pathogen *Fusarium oxysporum* (Sch1) f.sp lycopersici. Allelopathy Journal 31: 387–403.

[13] Xu N, Wei M, Wang C, Shi W, Tian FM, et al (2013) Composition of Welsh onion (*Allium fistulosum* L.) root exudates and their allelopathy on cucumber sprouts and *Fusarium oxysporum* f.sp cucumerinum. Allelopathy Journal 32: 243–256.

[14] Mangla S, Inderjit, Callaway RM (2008) Exotic invasive plant accumulates native soil pathogens which inhibit native plants. Journal of Ecology 96: 58–67.

[15] Malmstrom CM, McCullough AJ, Johnson HA, Newton LA, Borer ET (2005) Invasive annual grasses indirectly increase virus incidence in California native perennial bunchgrasses. Oecologia 145: 153–164. 15875144 10.1007/s00442-005-0099-z

[16] Eppinga MB, Rietkerk M, Dekker SC, De Ruiter PC (2006) Accumulation of local pathogens: a new hypothesis to explain exotic plant invasions. OIKOS 114: 168–176.

[17] Lodhi MAK, Killingbeck KT (1980) Allelopathic inhibition of nitrification and nitrifying bacteria in a ponderosa pine (*Pinus ponderosa* Dougl.) community. American Journal of Botany 67: 1423–1429.

[18] Lankau RA (2011) Intraspecific variation in allelochemistry determines an invasive species’ impact on soil microbial communities. Oecologia 165: 453–463. 20680644 10.1007/s00442-010-1736-8

[19] Kourtev PS, Ehrenfeld JG, Häggblom M (2002) Exotic plant species alter the microbial community structure and function in the soil. Ecology 83: 3152–3166.

[20] Zhang Q, Yang R, Tang J, Yang H, Hu S, et al (2010) Positive feedback between mycorrhizal fungi and plants influences plant invasion success and resistance to invasion. PLoS One 5: e12380. 20808770 10.1371/journal.pone.0012380 PMC2927435

[21] Reinhart KO, Callaway RM (2006) Soil biota and invasive plants. New Phytologist 170: 445–457. 16626467 10.1111/j.1469-8137.2006.01715.x

[22] Smith SE, Read DJ (1997) Mycorrhizal symbiosis. London, UK: Academic Press.

[23] Rillig MC (2004) Arbuscular mycorrhizae and terrestrial ecosystem processes. Ecology Letters 7: 740–754.

[24] Richardson D, Allsopp N, Antonio C, Milton S, Rejmanek M (2000) Plant invasions – the role of mutualisms. Biological Reviews 75: 65–93. 10740893 10.1017/s0006323199005435

[25] Pringle A, Bever JD, Gardes M, Parrent JL, Rillig MC, et al (2009) Mycorrhizal symbioses and plant invasions. Annual Review of Ecology, Evolution, and Systematics 40: 699–715.

[26] Marler MJ, Zabinski CA, Callaway RM (1999) Mycorrhizae indirectly enhance competitive effects of an invasive forb on a native bunchgrass. Ecology 80: 1180–1186.

[27] Carey EV, Marler MJ, Callaway RM (2004) Mycorrhizae transfer carbon from a native grass to an invasive weed,evidence from stable isotopes and physiology. Plant Ecology 172: 133–141.

[28] Stinson KA (2006) Invasive plant suppresses the growth of native tree seedlings by disrupting belowground mutualisms. PLoS BIOLOGY 4: e140. 16623597 10.1371/journal.pbio.0040140 PMC1440938

[29] Callaway RM (2008) Novel weapons: invasive plant suppresses fungal mutualists in America but not in its native Europe. Ecology 89: 1043–1055. 18481529 10.1890/07-0370.1

[30] Cantor A, Hale A, Aaron J, Traw MB, Kalisz S (2011) Low allelochemical concentrations detected in garlic mustard-invaded forest soils inhibit fungal growth and AMF spore germination. Biological Invasions 13: 3015–3025.

[31] Barto EK, Antunes PM, Stinson K, Koch AM, Klironomos JN, et al (2011) Differences in arbuscular mycorrhizal fungal communities associated with sugar maple seedlings in and outside of invaded garlic mustard forest patches. Biological Invasions 13: 2755–2762.

[32] Akiyama K, Matsuzaki K-I, Hayashi H (2005) Plant sesquiterpenes induce hyphal branching in arbuscular mycorrhizal fungi. Nature 435: 824–827. 15944706 10.1038/nature03608

[33] Buee M, Rossignol M, Jauneau A, Ranjeva R, Bécard G (2000) The pre-symbiotic growth of arbuscular mycorrhizal fungi is induced by a branching factor partially purified from plant root exudates. The American Phytopathological Society 13: 693–698. 10.1094/MPMI.2000.13.6.693 10830269

[34] Nagahashi G, Douds DD (2000) Partial separation of root exudate components and their effects upon the growth of germinated spores of AM fungi. Mycological Research 104: 1453–1464.

[35] Piotrowski JS, Morford SL, Rillig MC (2008) Inhibition of colonization by a native arbuscular mycorrhizal fungal community via *Populus trichocarpa* litter, litter extract, and soluble phenolic compounds. Soil Biology and Biochemistry 40: 709–717.

[36] Scervino JM, Ponce MA, Erra-Bassells R, Bompadre J, Vierheilig H, et al (2007) The effect of flavones and flavonols on colonization of tomato plants by arbuscular mycorrhizal fungi of the genera *Gigaspora* and *Glomus* . Canadian Journal of Microbiology 53: 702–709. 17668030 10.1139/W07-036

[37] Callaway RM, Aschehoug ET (2000) Invasive plants versus their new and old neighbors: a mechanism for exotic invasion. Science 290: 521–523. 11039934 10.1126/science.290.5491.521

[38] Weber E (1998) The dynamics of plant invasions: a case study of three exotic goldenrod species (*Solidago* L.) in Europe. Journal of Biogeography 25: 147–154.

[39] Jin L, Gu Y, Xiao M, Chen J, Li B (2004) The history of *Solidago canadensis* invasion and the development of its mycorrhizal associations in newly-reclaimed land. Functional Plant Biology 31: 979–986. 10.1071/FP04061 32688966

[40] Zhang Q, Yao L, Yang R, Yang X, Tang J, et al (2007) Potential allelopathic effects of an invasive species *Solidago canadensis* on the mycorrhizae of native plant species. Allelopathy Journal 20: 71–78.

[41] Mahall BE, Callaway RM (1992) Root communication mechanisms and intracommunity distributions of two Mojave desert shrubs. Ecology 73: 2145–2151.

[42] Ridenour WM, Callaway RM (2001) The relative importance of allelopathy in interference: the effects of an invasive weed on a native bunchgrass. Oecologia 126: 444–450. 28547460 10.1007/s004420000533

[43] Inderjit, Callaway RM (2003) Experimental designs for the study of allelopathy. Plant and Soil 256: 1–11.

[44] Lau JA, Puliafico KP, Kopshever JA, Steltzer H, Jarvis EP, et al (2008) Inference of allelopathy is complicated by effects of activated carbon on plant growth. New Phytologist 178: 412–423. 18208469 10.1111/j.1469-8137.2007.02360.x

[45] Gundale MJ, DeLuca TH (2006) Charcoal effects on soil solution chemistry and growth of *Koeleria macrantha* in the ponderosa pine/Douglas-fir ecosystem. Biology and Fertility of Soils 43: 303–311.

[46] Nelson DW, Sommers LE (1982) Total carbon, organic carbon, and organic matter, In: Rh, M., Dr, K. (Eds.), Methods of soil analysis, agronomy, part 2, 2nd. WI: ASA and SSSA, Madison, 539–577.

[47] Olsen SR, Cole CV, Watanabe FS, Dean LA (1954) Estimation of available phosphorus in soils by extraction with sodium bicarbonate. USDA circular, 939.

[48] Giovannetti M, Mosse B (1980) An evaluation of techniques for measuring vesicular arbuscular mycorrhizal infection in roots. New Phytologist 84: 489–500.

[49] Zhang S, Zhu W, Wang B, Tang J, Chen X (2011) Secondary metabolites from the invasive *Solidago canadensis* L. accumulation in soil and contribution to inhibition of soil pathogen *Pythium ultimum* . Applied Soil Ecology 48: 280–286.

[50] Hammer Ø, Harper DAT, Ryan PD (2001) PAST: paleontological statistics software package for education and data analysis. Palaeontologia Electronica 4: 1–9.

[51] Tang J, Zhang Q, Yang R, Chen X (2009) Effects of exotic plant *Solidago canadensis* L. on local arbuscular mycorrhizal fungi. Bulletin of Science and Technology 25: 233–237.

[52] Gerdemann J, Nicolson TH (1963) Spores of mycorrhizal *Endogone* species extracted from soil by wet sieving and decanting. Transactions of the British Mycological Society 46: 235–244.

[53] Scheublin TR, Van Logtestijn RSP, Van Der Heijden MGA (2007) Presence and identity of arbuscular mycorrhizal fungi influence competitive interactions between plant species. Journal of Ecology 95: 631–638.

[54] Lehmann J Jr. JPdS, Steiner C, Nehls T, Zech W, et al (2003) Nutrient availability and leaching in an archaeological anthrosol and a ferralsol of the central amazon basin: fertilizer, manure and charcoal amendments. Plant and Soil 249: 343–357.

[55] DeLuca TH, MacKenzie MD, Gundale MJ, Holben WE (2006) Wildfire-produced charcoal directly influences nitrogen cycling in ponderosa pine forests. Soil Science Society of America Journal 70: 448–453.

[56] Matsubara Y, Hasegawa N, Fukui H (2002) Incidence of Fusarium root rot in asparagus seedlings infected with arbuscular mycorrhizal fungus as affected by several soil amendments. Journal of the Japanese Society for Horticultural Science 71: 370–374.

[57] Besserer A, Puech-Pagès V, Kiefer P, Gomez-Roldan V, Jauneau A, et al (2006) Strigolactones stimulate arbuscular mycorrhizal fungi by activating mitochondria. PLoS BIOLOGY 4: e226. 16787107 10.1371/journal.pbio.0040226 PMC1481526

[58] Harborne JrB, Williams CA (2000) Advances in flavonoid research since 1992. Phytochemistry 55: 481–504. 11130659 10.1016/s0031-9422(00)00235-1

[59] Mert-Türk F (2006) Saponins versus plant fungal pathogens. Journal of Cell and Molecular Biology 5: 13–17.

[60] Kim YO, Lee EJ (2010) Comparison of phenolic compounds and the effects of invasive and native species in East Asia: support for the novel weapons hypothesis. Ecological Research 26: 87–94.

[61] Perry LG, Thelen GC, Ridenour WM, Weir TL, Callaway RM, et al (2005) Dual role for an allelochemical: (±)-catechin from *Centaurea maculosa* root exudates regulates conspecific seedling establishment. Journal of Ecology 93: 1126–1135.

[62] Cappuccino N, Arnason JT (2006) Novel chemistry of invasive exotic plants. Biology Letters 2: 189–193. 17148359 10.1098/rsbl.2005.0433 PMC1618907

