# Supplementary figures and images for: An Invasive Plant Promotes Its Arbuscular Mycorrhizal Symbioses and Competitiveness through Its Secondary Metabolites: Indirect Evidence from Activated Carbon
Source: PLoS One. 2014 May 9;9(5):e97163. doi: 10.1371/journal.pone.0097163 (PMC4016281; doi:10.1371/journal.pone.0097163)

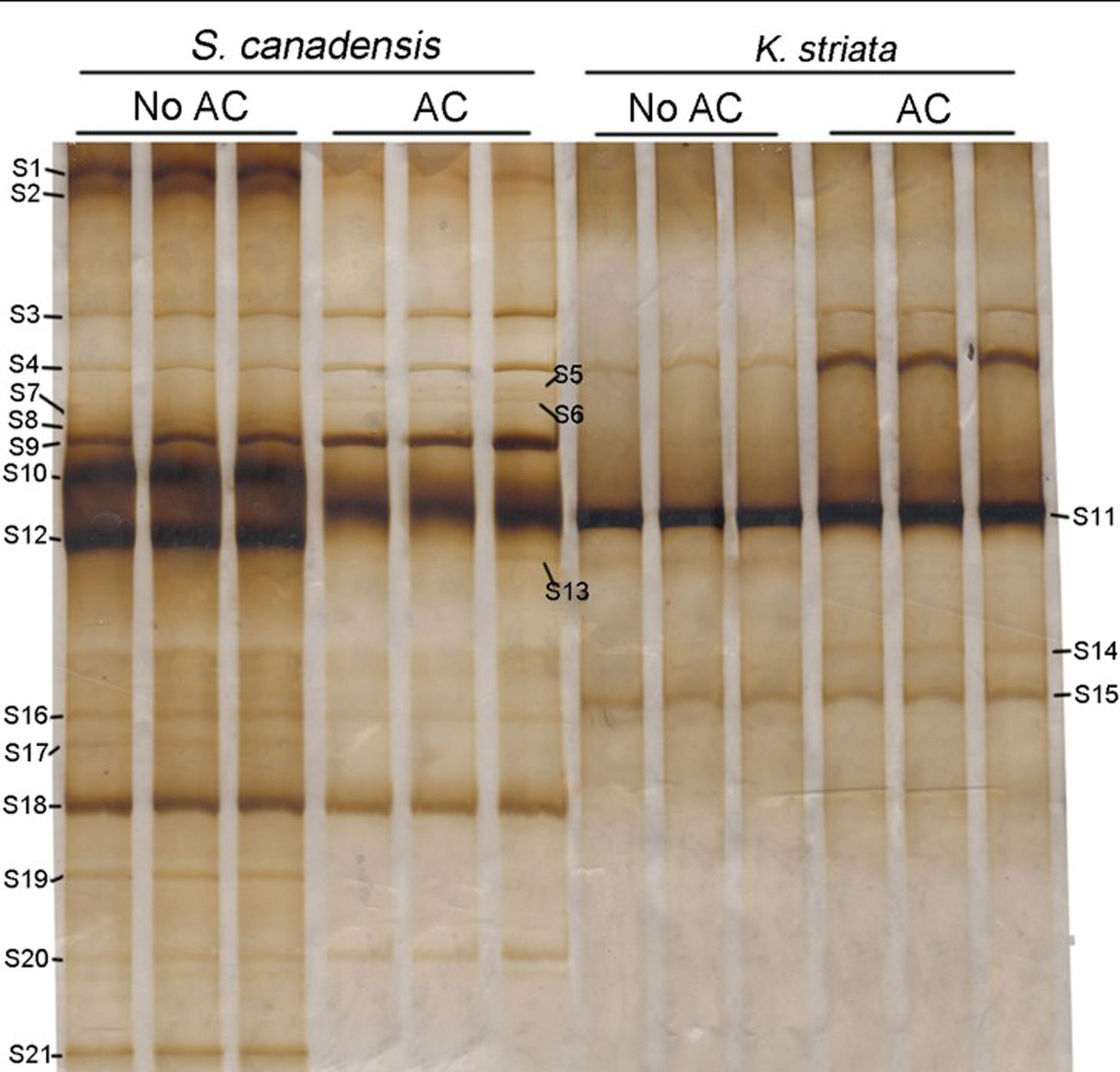

Supplement: Figure S1 — Effect of AC on DGGE pattern of AMF communities in experiment 1. DGGE pattern of 18S rDNA fragments of AMF in roots of S. canadensis and K. striata as affected by addition of activated carbon (AC and No AC) in experiment 1. (TIF) [file pone.0097163.s001.tif]

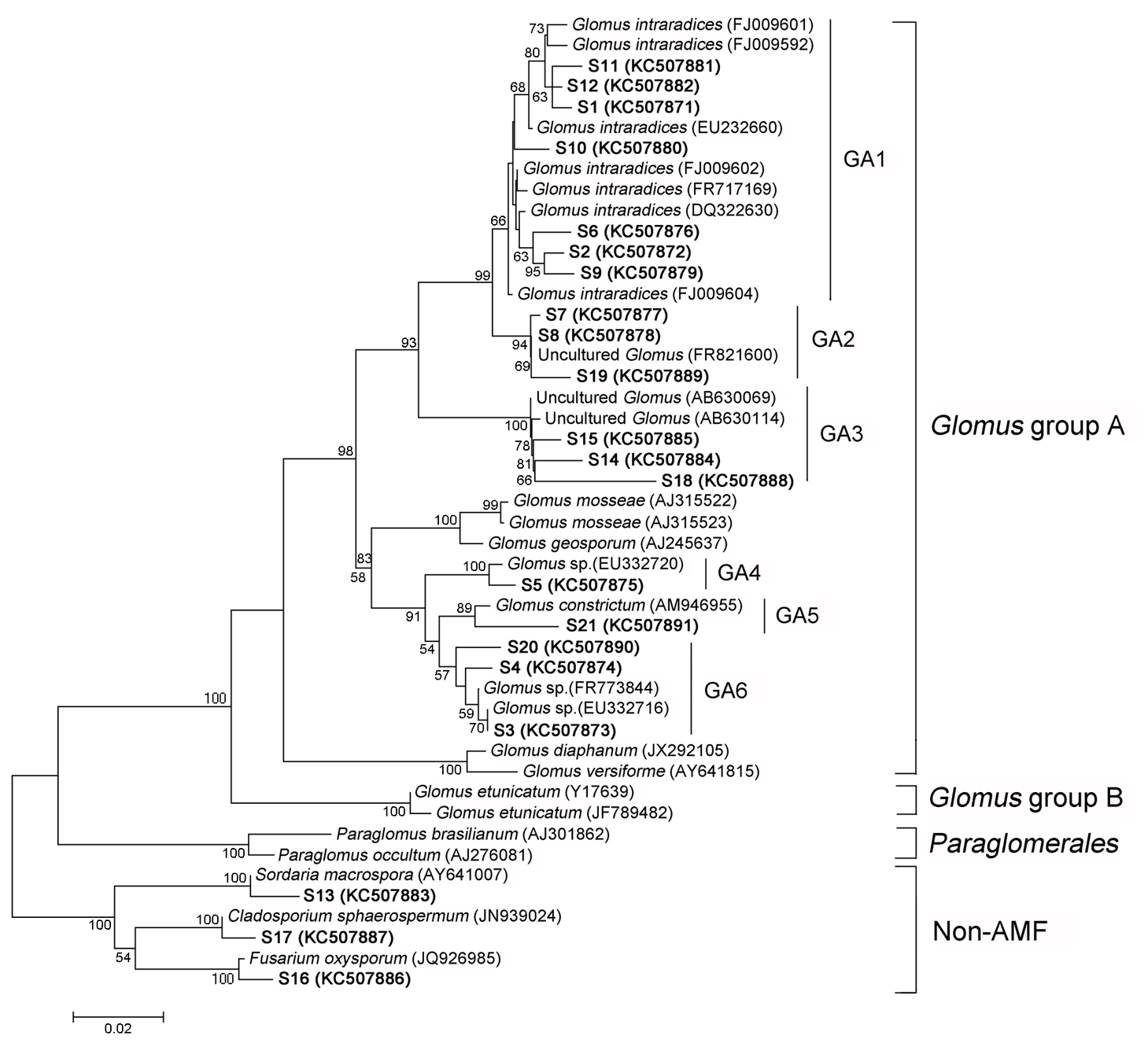

Supplement: Figure S2 — Phylogenetic tree of AMF communities in experiment 1. Neighbor-joining phylogenetic tree based on partial SSU rRNA gene sequences of all identified AMF and referenced sequences in the root samples. Numbers above branches indicate bootstrap values from 1,000 replicates. The internal identification number represents sequences retrieved from specific DGGE profile bands. A total of 21 partial SSU rRNA sequences were thus obtained in the present study. They are shown in bold and labeled with the GeneBank database accession numbers (i.e., KC507871–KC507891). Sequence groups (GA1, GA2, etc.) identify distinct clusters of sequences with similarity ≥99%. (TIF) [file pone.0097163.s002.tif]
