# Supplementary material for: An Invasive Plant Promotes Its Arbuscular Mycorrhizal Symbioses and Competitiveness through Its Secondary Metabolites: Indirect Evidence from Activated Carbon
Source: PLoS One. 2014 May 9;9(5):e97163. doi: 10.1371/journal.pone.0097163 (PMC4016281; doi:10.1371/journal.pone.0097163)
Supplement: Methods S1 — Methods used for DNA isolation, PCR amplification, DGGE, cloning, and sequencing analysis of AMF. (DOC) [file pone.0097163.s003.doc]

**Method S1. Methods used for AMF community analysis in experiment 1.** Methods used for DNA isolation, PCR amplification, DGGE, cloning, and sequencing analysis of AMF

*DNA isolation and PCR amplification*

Genomic DNA was extracted from root samples using a DNA Extraction Kit and following the manufacturer’s protocol (Axygen Biosciences, China). Isolated genomic DNA was subjected to nested PCR with primers GeoA2/Geo11 [1] and AM1/GC-NS31 [2,3]. Thermocycling program and conditions for the first PCR with primers GeoA2/Geo11 were 94°C for 2 min; followed by 30 cycles at 94°C for 30 s, 59°C for 1 min, and 72°C for 2 min; and a final extension at 72°C for 10 min. The 25-μl reaction volume contained 2.5 μl of 10× buffer, 2 μl of dNTP (2.5 mM), 0.5 μl of each primer (10 pmol), 2 μl of template, 0.25 μl of Taq polymerase (Takara, Japan), and ddH_2_O. The 1800-bp PCR products were analyzed by agarose gel electrophoresis (1.0% (w/v) agarose, 120 V, 45 min) and ethidium bromide staining. The first-step PCR products were diluted 1:100, and 2 μl of this dilution was used as a template for the second PCR. The second PCR used identical reaction conditions as the first PCR with the primers AM1/GC-NS31 and the following program: 94°C for 2 min; followed by 30 cycles at 94°C for 30 s, 67°C for 1 min, and 72°C for 2 min; and a final extension at 72°C for 10 min. The nested PCR products were examined on an agarose gel as described above.

*DGGE analysis*

PCR products were used for DGGE analysis with the Decode™ Universal Mutation Detection System (Bio-Rad, Hercules, CA, USA) as described by Kowalchuk et al. [3]. Electrophoresis was run at 150 V and 60°C for 6 h. Gels were stained using silver [4], and gel images were captured digitally using a scanner (Epson, Japan). The DGGE band pattern and intensity were analyzed by Quantity One Software (Bio-Rad, Hercules, CA, USA).

*Cloning and sequencing*

To obtain sequences from DGGE bands, each DGGE band was excised. Then the DNA in the band was eluted and reamplified with primers AM1/NS31 (no GC-clamp added) following the second PCR procedure described above. DNA fragments of expected length (about 550 bp) were purified using the Gel Clean kit (Axygen Biosciences, China) according to the manufacturer’s instructions. Purified PCR products were digested with the restriction enzymes HinfI and AluI (Takara, Japan) to confirm that bands with the same mobility contained the same sequence. One purified PCR product from each RFLP/mobility group was selected randomly, ligated into pGEM-T (Promega), and cloned into *Escherichia coli* DH5α according to the manufacturer’s recommended protocol. The transformed cells were plated onto LB (Luria-Bertani) medium (1.0% Bacto-Tryptone, 0.5% Bacto-yeast extract, 1.0% NaCl, 1.5% Bacto-agar, pH 7.0) containing ampicillin (50 μg ml^−1^) and X-Gal (0.1 mM), and white-coloured recombinant colonies were identified. The presence of inserts of the expected size was confirmed by PCR using the primers AM1/NS31 (PCR conditions as described above). Reconfirmed clones were sequenced by the Shanghai Sangon Biological Engineering Technology & Services Co., Ltd.

**References**

1. Schwarzott D, Schüßler A (2001) A simple and reliable method for SSU rRNA gene DNA extraction, amplification, and cloning from single AM fungal spores. Mycorrhiza 10:203-207.

2. Helgason T, Daniell T, Husband R, Fitter A, Young J (1998) Ploughing up the wood-wide web. Nature 394:431-431.

3. Kowalchuk GA, De Souza FA, van Veen JA (2002) Community analysis of arbuscular mycorrhizal fungi associated with *Ammophila arenaria* in Dutch coastal sand dunes. Mol Ecol 11:571-581.

4. Sanguinetti C, Dias NE, Simpson A (1994) Rapid silver staining and recovery of PCR products separated on polyacrylamide gels. Biotechniques 17:914.
